# Occlusion and Disocclusion Time Changes in Single Unit Crowns Designed by Functional Generated Path Technique: A Randomised Clinical Trial

**DOI:** 10.1038/s41598-017-00408-0

**Published:** 2017-03-24

**Authors:** Ping-ting Lin, Yang Jiao, San-jun Zhao, Fu Wang, Ling Li, Fan Yu, Min Tian, Hao-han Yu, Ji-hua Chen

**Affiliations:** 1 State Key Laboratory of Military Stomatology & National Clinical Research Centre for Oral Disease & Shaanxi key Laboratory of Oral Disease, Department of Prosthodontics, School of Stomatology, Fourth Military Medical University, Xi’an, 710032 P.R. China; 2 Department of Stomatology, PLA Army General Hospital, Beijing, 100700 P. R. China

## Abstract

Although it is believed that implementation of the functional generated path (FGP) technique can facilitate occlusal surface design for restorations, it has not been objectively compared *in situ* with the conventional fabrication yet. Therefore, in the present study, a single-blind crossover clinical trial was conducted using T-scan to compare changes in occlusion time (OT) and disocclusion time (DT) of single posterior artificial crowns designed differently using FGP technique (FGP), average-value FGP technique (AVR) and conventional fabrication (CON). Each of the 10 participants took part in the study tried three artificial crowns in different sequences according to a computer generated randomization list. The results objectively revealed that changes in OT and DT were significantly smaller for FGP than CON (P < 0.05) and considerably smaller for AVR than CON, respectively. The subjective feedback and the occlusal adjusting time were better and shorter for FGP and AVR than CON (P < 0.05). No harm to the participants occurred. Overall, FGP is an efficient technique showing more physiological harmonious relationship with the articulating system.

## Introduction

Occlusion and occlusal surface are important subjects in daily dental practice^1^. When performing single crown restoration in patients, it is important that the crown contributes to harmonious function^2, 3^. Traditionally, crown occlusal surface is designed based on static relationship^4^. However, when patient’s dynamic occlusion was introduced on try-in visit, occlusal interference usually occurs^5–7^. To solve the problem, using articulator for accurate measurement and registration of jaw movements and temporomandibular joint (TMJ) parameters is essential. Nonetheless, the procedure could be time-consuming. Besides, how much individual registration is actually needed in order to keep occlusal interference within acceptable tolerance limits is still under debate^8, 9^.

Undoubtedly, the most accurate articulator is the patient him/herself. Meyer *et al*. first proposed the concept of functional generated path (FGP) in 1959 by recording the occlusal path of the teeth during movement directly in the patient’s mouth^10^. Thereafter, different materials and methods had been applied in FGP fixed prosthodontics^5, 6, 11–19^. Some researchers even tried to integrate the concept into CAD/CAM manufacturing^5–7, 12–18^. For single unit crowns, Mehl^3^ reviewed several related studies and deduced that FGP could be simulated by average-setting articulator in most cases, saving a great deal of time and devices in crown fabrication.

Numerous reports have illustrated that FGP has drawn more and more attentions and is useful for fixed prosthodontics. However, very few reports compared FGP technique and conventional fabrication^6, 11^, and none of them was an objective comparison *in situ*. T-scan system is among one of the most frequently used computerized analyzing systems to objectively assess occlusal equilibration^20–24^. The system records relative force values and objectively quantifies occlusal balance by displaying numerical values for occlusion and disocclusion times. Occlusion time (OT) is defined as the time from the first contact of occluding teeth to maximum intercuspation, whereas disocclusion time (DT) is defined as the time from maximum intercuspation to complete disocclusion during lateral movement^25, 26^. OT and DT are helpful in occlusal adjustment and linking the occlusion with other elements of the articulatory system^21, 22, 27^.

The purpose of this study was to compare OT and DT changes of the crown designed using FGP and conventional fabrication. Our null hypothesis was there were no significant differences between OT and DT of the crown designed using FGP technique and those using conventional fabrication.

## Materials and Methods

A single-blind crossover clinical trial was adopted. The evaluation was carried out among single crowns designed differently using FGP technique (FGP), average-value FGP technique (AVR) and conventional fabrication (CON) in a sequence determined by a computer-generated list of random numbers. The study sample included 10 participants (5 men and 5 women, aged from 19 to 65 years old, with an average of 44.2 years old) selected from the Department of Prosthodontics at Stomatological Hospital of Fourth Military Medical University from December 2015 to March 2016. Power analysis was conducted using software G*Power, v. 3.1.9.2 (University of Kiel). Use of an alpha value of 0.05, a sample size of 10 was found to yield a power of 0.8. All participants were given detailed information about the study and singed written informed consent. The study protocol was reviewed and approved by the Institutional Review Board of Stomatological Hospital of Fourth Military Medical University (approval number: IRB-REV-2015039; Supplementary Fig. 1). It was further registered under protocol ID ClinicalTrials.gov NCT02609178 (date of registration:05/11/2015) according to the CONSORT 2010 statement. The methods employed were performed in accordance with the approved guidelines.

All participants had Angle class I jaw relationship with a single tooth needs crown restoration in the posterior quadrant (3 premolars and 7 molars). The tooth’s antagonist was purely natural or had received minimum restorations that would not significantly change the occlusal morphology. In other words, the antagonist could have received restorations on the surfaces other than the occlusal surface. If the restorations were on the occlusal surface, they should not cover the cusps^28, 29^. Exclusion criteria were the presence of missing tooth, moderate or severe periodontitis, temporomandibular joint disease, parafunctional movements or orthodontic treatment history.

**Figure 1:**
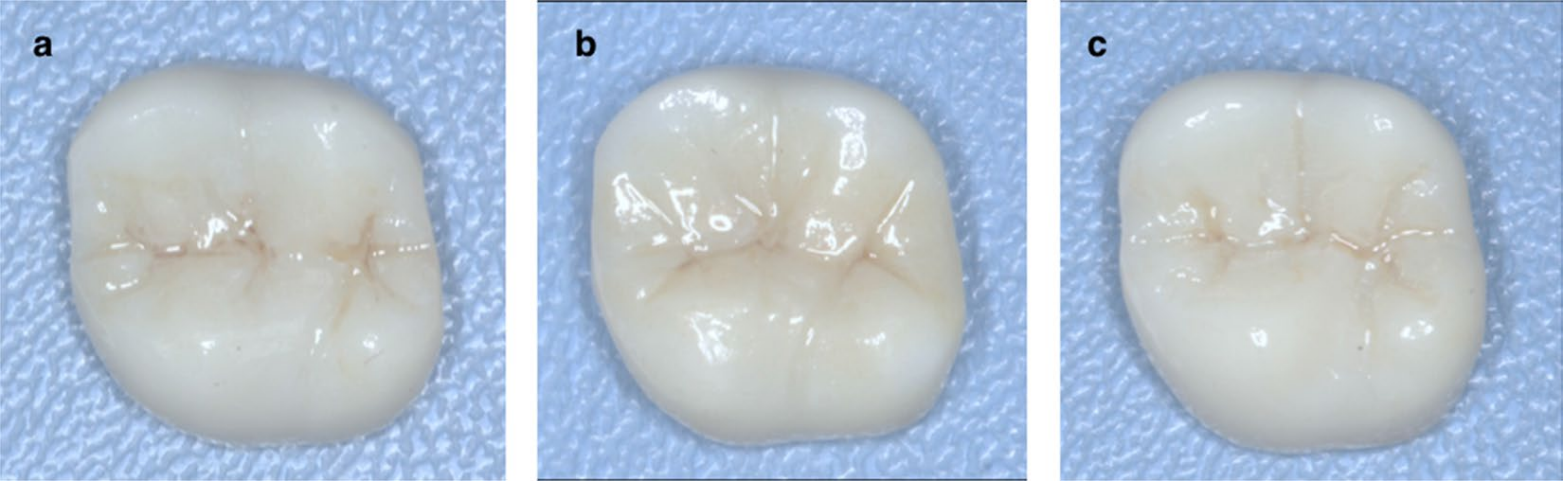
Example of the crowns designed differently in the study. (**a**) The ceramic crown designed by FGP technique (FGP). The crown was a copy of the interim made in the participant’s mouth by the FGP technique. (**b**) The ceramic crown designed with the average-value virtual articulator (AVR). (**c**) The ceramic crown designed by conventional fabrication (CON), which was fabricated by the technician from his own experience in the CAD/CAM software.

All the teeth were prepared by 1 of 3 experienced practitioners (S.J.Z, F.W and L.L) under standard recommended preparation guidelines of 1.5 to 2.0 mm occlusal reduction, and 1.0 to 1.5 mm axial reduction with a deep chamfer margin circumferentially. Margin placement was designated at no more than 0.5 mm subgingivally. The impressions were made of A-silicones using two-phase impression technique (Silagum, DMG, Germany). The interim of the prepared teeth was fabricated in the laboratory using CAD/CAM (Dental system, 3Shape, Denmark; D710, Wieland, Germany) with 1 mm occlusal clearance. Participants were recalled for the first visit to perform general FGP technique. Light-cured resin (Clip F, VOCO, Germany) was applied onto the occlusal surface until the interim was properly fit with 1 mm occlusal clearance. Afterwards, the participants were instructed to close in maximum intercuspation position and then to perform right lateral, left lateral and protrusive movements in succession ending at maximum intercuspation position. After excess resin was trimmed off, the resin was fully polymerized under the manufacturer's instruction. The ICP contacts were marked using a 40 μm articulating paper (Arti-Check BK09, Bausch, Germany). Then a 12 μm shimstock (Arti-Fol BK31, Bausch, Germany) was used while instructing the participants to perform the eccentric movements. All eccentric interferences were eliminated and care was taken not to grind the ICP contacts registered in blue. The interim was then sent back to the laboratory to make the final restoration by duplicating its occlusal scheme (marked as FGP) (Dental System, 3Shape, Denmark; D710, Wieland, Germany). Meanwhile, two more final restorations of the same prepared tooth were made using average-value FGP technique (marked as AVR) and conventional fabrication (marked as CON), respectively (Dental system, 3Shape, Denmark; D710, Wieland, Germany). AVR was designed with the help of the virtual articulator to set the average values, as Mehl^5^ described. The average values adopted in the study were 30° for the angles of the sagittal condyle, 15° for the lateral Bennett angle, and 30° for the incisal path, respectively^6^. CON was designed purely from the technician’s experience. To avoid error, all the zirconia crowns were designed by the same technician and fabricated in fully anatomical form using Upcera blocks (Upcera, Upcera Dental, China) without using veneering porcelain (Fig. 1).

Each participant’s 3 crowns were packed separately with notes illustrating their try-in order following simple randomization procedures (computerized random numbers) in advance. On the try-in visit, each participant tried in the three crowns in corresponding order. First, OT and DT were recorded before try-in of any crowns as the baseline. Then the same participant started to try in each of the 3 crowns, and OT and DT were recorded again when the crown was seated before occlusal adjustments. The participant was at the same time inquired to grade their feeling towards the occlusal interference of the crown using Likert’s 3-point scale^11^ (Table 1). Adjustments were done by the same clinician (P. T. L) to remove premature occlusal contacts and occlusal interference, and the adjusting time for occlusal surface was recorded. A 5-minute interval was given to each participant between try-in of each crown. At last, the best fitting crown was chosen by the participant and cemented using glass ionomer cement (Fuji 1, GC, Japan).

**Table 1:** Likert’s 3-point scale.

Score	Feeling
0	No interference
1	Moderate interference
2	High interference

In the study, OT and DT examinations were carried out using T-scan system (T-scan III, Teckscan, USA) by the same examiner (P. T. L). The size of the 100-μm-thick sensor (large or small) was chosen to suit the participant’s dental arch. Prior to any occlusal data acquisition, a proper sensitivity range was established^25, 30^ according to the manufacturer’s recommendation, and the sensor conditioning procedures of 2 to 4 test closures^27, 31^ were performed for each participant.

For all scanning procedures, participants were asked to sit in a relaxed upright position in the dental chair. The sensor was held consistently at the same position with respect to the teeth and aligned parallel to the occlusal plane and centered on the midline between the central incisors^31^. The same sensor was used for each participant throughout the try-in visit.

When recording OT and DT, participants were asked to 1) occlude on the sensor in centric occlusion with normal pressure until maximum intercuspation, 2) hold their teeth together for a period of 1 to 3 seconds, 3) start a protrusion from that completely intercuspated position, and 4) disocclude^31^. This procedure was repeated 3 times. Changes in OT and DT (ΔOT and ΔDT) were calculated by the following formulas:

1 $${\rm{\Delta }}\mathrm{OT}={\rm{mean}}\,{\rm{OT}}\,({\rm{seated}}\,{\rm{without}}\,{\rm{adjustment}})-{\rm{mean}}\,{\rm{OT}}\,({\rm{baseline}})$$

2 $${\rm{\Delta }}\mathrm{DT}={\rm{mean}}\,{\rm{DT}}\,({\rm{seated}}\,{\rm{without}}\,{\rm{adjustment}})-{\rm{mean}}\,{\rm{DT}}\,({\rm{baseline}})$$

The mean OT, DT, ΔOT and ΔDT of all the participants were calculated and statistically analyzed using SPSS 19.0 for Windows. All data were expressed as mean ± standard deviation. ΔOT and ΔDT were first tested by the Kolmogorov-Smirnov test of normality and then by the test for the homogeneity of variance. The results showed most of the data were normally distributed, thus one-way ANOVA (α = 0.05) was conducted to compared ΔOT and ΔDT among the 3 different crown designs – FGP, AVR and CON. The Kruskal-Wallis test (α = 0.05) was used to compare Likert’s 3-point scale and adjusting time among the 3 different crown designs.

## Results

**Figure 2:**
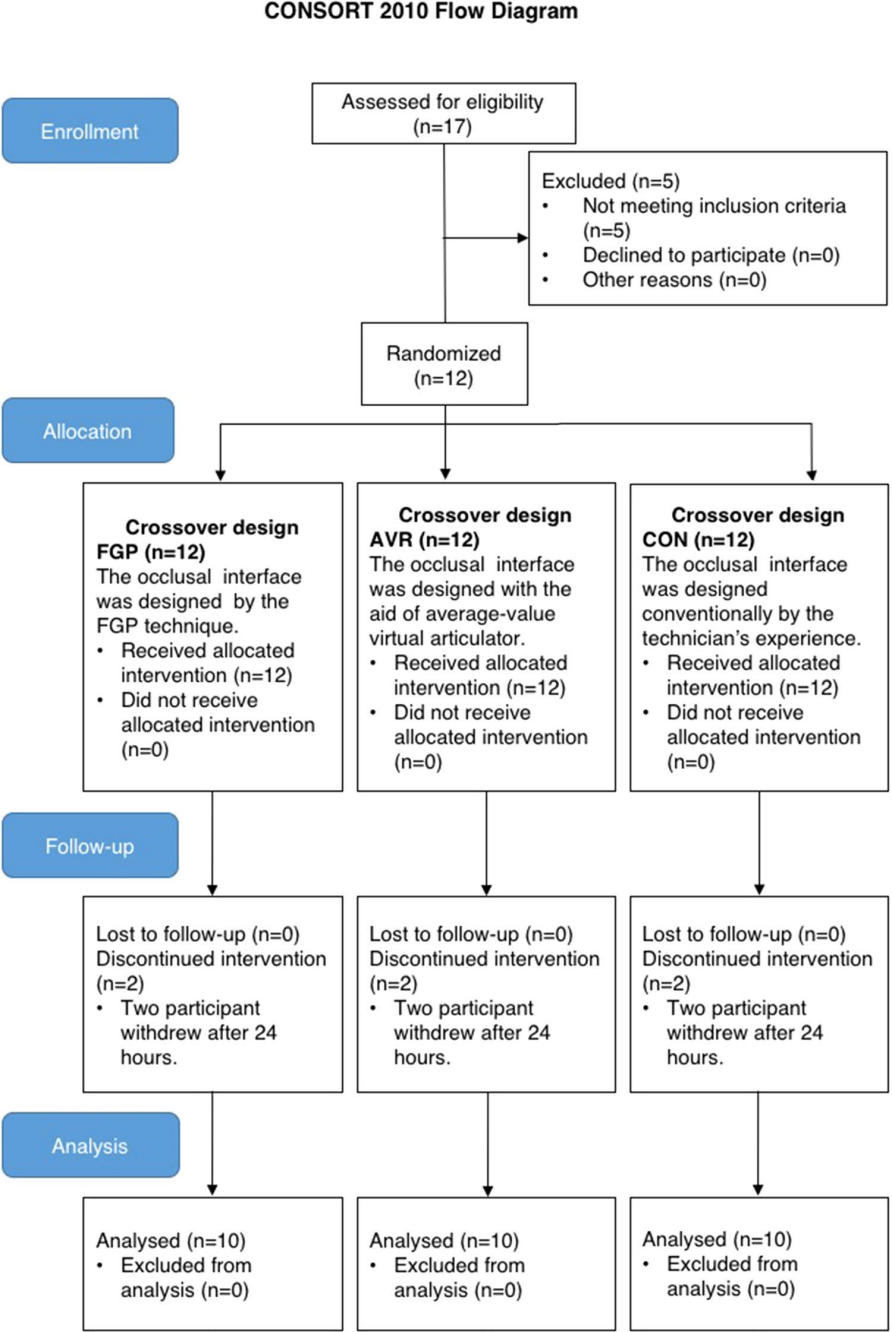
CONSORT flow diagram of subject randomization and selection criteria.

**Figure 3:**
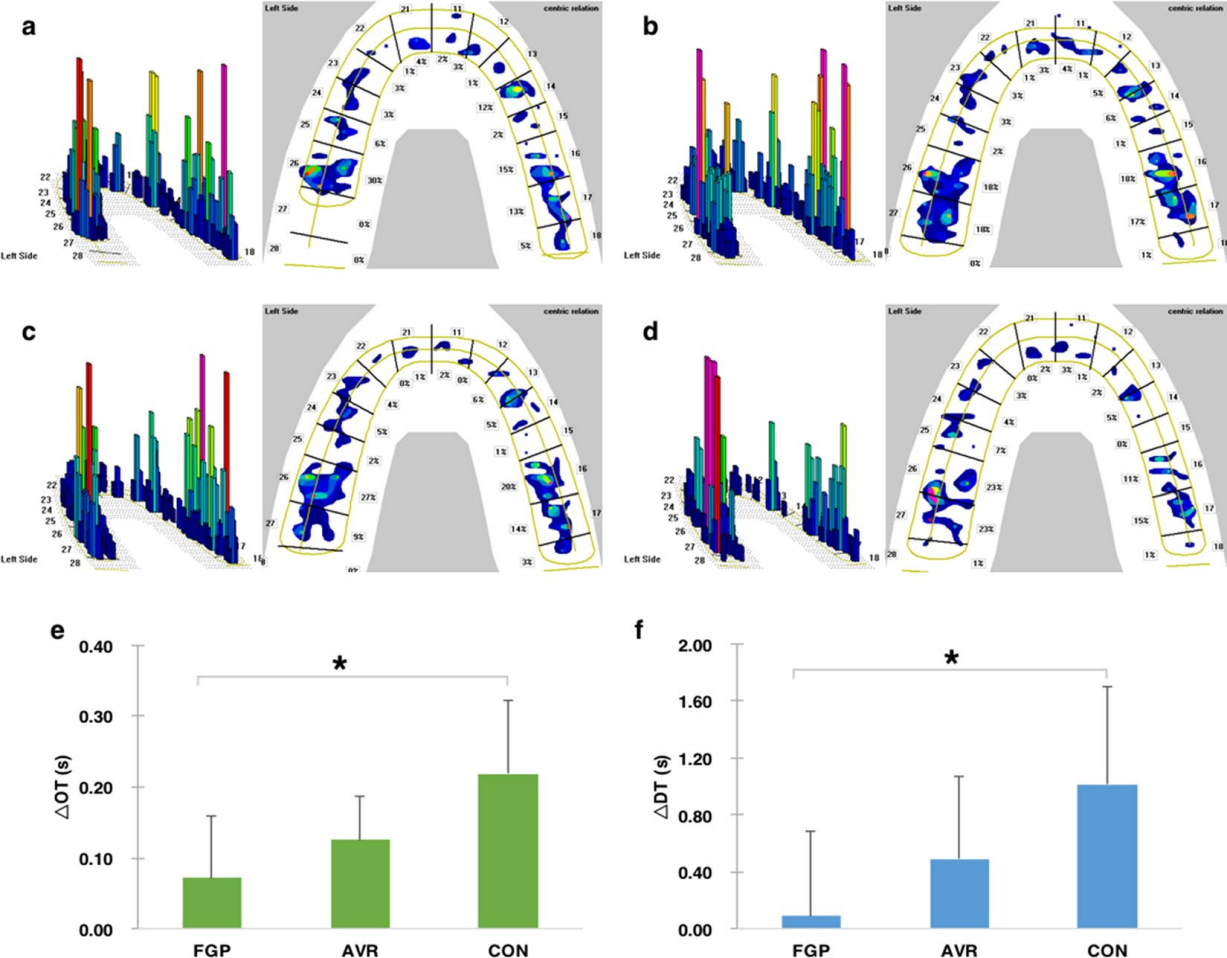
T-scan measurements of the study. (**a**–**d**) Example of the 2-D and 3-D results of the T-scan measurement. The movie was advanced to the MA frame in which maximum intercuspation occurred. (**a**) The baseline image. (**b**) Try-in of FGP. The force loaded on tooth 26 at baseline distributed equally on tooth 26 and 27 while maintaining almost the same occlusal balance to the baseline. (**c**) Try-in of AVR. Tooth 27 shared part of the force loaded on tooth 26 at baseline. Occlusal balance was maintained. (**d**) Try-in of CON. Occlusal balance was broke. Force concentration area was detected on tooth 27. (**e**) Result of the difference among FGP, AVR and CON in ∆OT. Significant smaller changes were observed between FGP and CON. (**f**) Result of the difference among FGP, AVR and CON in ∆DT. Significant smaller changes were observed between FGP and CON.

**Figure 4:**
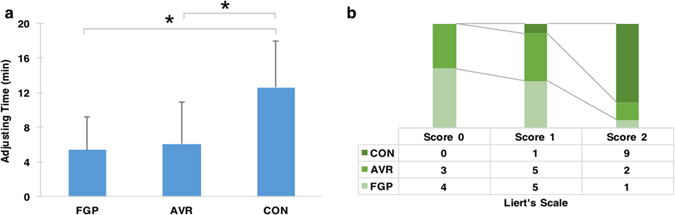
Secondary outcomes of the study. (**a**) Result of time used in adjusting FGP, AVR and CON. FGP and AVR needed less time than CON. (**b**) Result of the distribution difference among FGP, AVR and CON in Likert’s scale. Participants felt better with FGP and AVR crown than CON.

A CONSORT flow diagram illustrating subject flow during the clinical trial is presented in Fig. 2. All the experimental teeth had received root canal treatment before preparation. At last, 9 participants chose to take FGP and the other one chose AVR. No adverse events were reported. OT was 0.47 ± 0.57 s, 0.52 ± 0.58 s, 0.58 ± 0.55 s and 0.67 ± 0.56 s for baseline, FGP, AVR and CON, respectively, and DT was 0.89 ± 1.30 s, 0.98 ± 1.11 s, 1.38 ± 1.82 s and 1.91 ± 1.79 s for baseline, FGP, AVR and CON, respectively. Changes in OT were calculated as 0.07 ± 0.09 s, 0.13 ± 0.06 s and 0.22 ± 0.10 s for FGP, AVR and CON, respectively, and changes in DT were 0.09 ± 0.51 s, 0.49 ± 0.58 s and 1.02 ± 0.69 s for FGP, AVR and CON, respectively. Statistical analysis revealed significant smaller changes in OT and DT in FGP than in CON (P = 0.002 for OT; P = 0.005 for DT). Although AVR showed smaller OT and DT changes than CON, the difference was not statistically significant (Fig. 3). In addition, the average time needed for adjustment was 5.4 ± 3.8 min, 6.1 ± 4.8 min and 12.6 ± 5.3 min for FGP, AVR and CON, respectively. From the participants’ perspective, a significant better feedback was received from both FGP (P = 0.001) and AVR (P = 0.002) in try-in procedure (Fig. 4a). Statistical analysis showed both FGP (P = 0.01) and AVR (P = 0.029) crowns could significantly save time in occlusal adjustment, compared with CON (Fig. 4b).

## Discussion

Concept of FGP has long been adopted in fixed prosthodontic practice. In traditional lost-wax technique, FGP could be employed with double-casting technique. Previous studies have confirmed the efficiency and a better patients’ satisfaction of double-casted FGP crown in adjusting occlusal surface^11^. In the digital process, FGP has been integrated into the workflow of virtual articulator with the help of individual registrations of centric and eccentric jaw position^12–18^. Some researchers even developed scanning devices to aid in recording the occlusal movement path directly in patients’ mouth^6, 7^. However, these devices still need time to popularize. A simulation study on dental models compared the morphological difference of crowns designed using FGP, AVR and CON and found that crowns prepared using FGP and AVR had more stabilized occlusion than those prepared using CON^6^. However, no clinical study compared objectively between FGP technique and conventional fabrication *in situ* so far.

Occlusion is hard to be evaluated due to lack of available gold standard. For simple restorations such as single unit crown, a successful occlusal surface design should not significantly change the patients’ occlusion^32^. Stated by the guidance for occlusal adjustment of simple restoration, occlusal adjustment should apply to rectify the contact points of the adjacent teeth back to the state when no crown has been worn^32, 33^.

However, conventional ways of assessing occlusal contacts encounter their own limits in practical application. Although articulating paper has been most commonly used, its marks would be affected by its width, and the salivary impregnation can diffuse the paper ink, leading to false positives^34–36^. Transillumination of silicon bite registration can overcome the problems of articulating paper, but the result depends much on the orientation of the light resource and would be affected by the filler in the bite registration material^37, 38^.

The T-scan was adopted in the present study to detect occlusal contacts because it could not only locate premature contacts and occlusal interference more clearly^31, 39^, but also relate occlusion to other elements of articulatory system simply through OT and DT. OT is directly related with patients’ occlusal contact pattern^21^ and has been considered as a capable description of occlusion^22, 40^, whereas, DT could relate tooth contacts to muscle activity^26^. Abnormalities in DT would result in change of muscle activity, thus facilitating the occurrence of TMD^23^. According to the manufacturer, OT is recommended as less than 0.2 s, and DT less than 0.4 s^25, 26^. However, in a survey conducted by Haralur *et al*.^20^, the average OT and DT in normal dentate subjects with healthy TMJs were 0.69 s and 0.79 s respectively. Another survey performed by Ma *et al*.^41^ in Chinese population with Angle class I relationship and healthy TMJs showed an average of 0.34 s for OT and 1.00 s for DT. Also, in the present study, the average OT and DT at baseline were 0.47 s and 0.89 s, respectively, which were between the results obtained by Haralur *et al*. and Ma *et al*., but still much longer than the recommended values. These discrepancies may be probably explained by individual difference. Therefore, in the study we calculated ΔOT and ΔDT as the main indexes. On the other hand, among other 3 pairs of OT and DT recorded before adjustments were made, the average OT values of all groups fell within the range between Haralur *et al.* and Ma *et al*., but only the average DT values of FGP group fell within the range between Haralur *et al*. and Ma *et al*. The phenomenon indicated that more physiologic harmonious function might be achieved by FGP than other two kinds of crowns. The results of ΔOT and ΔDT rejected the null hypothesis and revealed that ΔOT and ΔDT were significantly smaller in FGP than CON, but the difference between AVR and CON was not significant. Moreover, the results of subjective evaluation of patients’ feedback and adjusting time exhibited more comfortable feeling and less chair-side time in FGP and AVR, compared with CON, which was consistent with the result of a previous study carried out by Memon^11^. Moreover, in another study performed by Olthoff *et al*.^6^, larger interocclusal distance was found in FGP and AVR than CON. These results similarly indicated easier occlusal adjustment of FGP and AVR.

To ensure valid and reliable results, several measures were considered. First, the present study was designed as a crossover clinical trial to exclude individual variations and 5-minute washout time was provided to eliminate the effect between each crown. Second, the occlusal adjustment and the recording of T-scan were carried out by the same examiner (P. T. L) to exclude interexaminer variations because no study has investigated the influence of operator on the values recorded by T-scan^42^. Furthermore, considering an increasingly significant relationship was reported between the sagittal plan head-neck posture and initial occlusal contacts for patients over the age of 30^43^, all the occlusal adjustment procedures were done with participants sitting in a relaxed upright position. As instructed by the manufacturer, the sensors of T-scan can be used up to 15 to 25 times^44^. Therefore, for each participant, the same sensor was used throughout the try-in visit to exclude intersensor variability.

The authors acknowledged that the present study did not cover all cohorts who needed single unit crown restoration. Although FGP technique generally showed good results, FGP crowns were not easy to fabricate and needed to be further examined in more follow-ups. Therefore, it is necessary to further simplify the technique.

## Conclusions

Within the limitations of the present study, FGP single crown showed significantly smaller changes in OT and DT than those from conventional single crowns, thus significantly improving patients’ satisfaction and reducing time for adjusting occlusion.
